# Neurodevelopmental Deceleration by Urban Fine Particles from Different Emission Sources: A Longitudinal Observational Study

**DOI:** 10.1289/EHP209

**Published:** 2016-04-29

**Authors:** Xavier Basagaña, Mikel Esnaola, Ioar Rivas, Fulvio Amato, Mar Alvarez-Pedrerol, Joan Forns, Mònica López-Vicente, Jesús Pujol, Mark Nieuwenhuijsen, Xavier Querol, Jordi Sunyer

**Affiliations:** 1 Centre for Research in Environmental Epidemiology (CREAL), Barcelona, Catalonia, Spain; 2 Universitat Pompeu Fabra (UPF), Barcelona, Catalonia, Spain; 3 CIBER Epidemiología y Salud Pública (CIBERESP), Spain; 4 Institute of Environmental Assessment and Water Research (IDAEA-CSIC), Barcelona, Catalonia, Spain; 5 Department of Genes and Environment, Division of Epidemiology, Norwegian Institute of Public Health, Oslo, Norway; 6 MRI Research Unit, CRC Mar, Hospital del Mar, Barcelona, Spain; 7 Centro Investigación Biomédica en Red de Salud Mental (CIBERSAM G21), Barcelona, Spain; 8 IMIM (Hospital del Mar Medical Research Institute), Barcelona, Catalonia, Spain

## Abstract

**Background:**

A few studies have reported associations between traffic-related air pollution exposure at schools and cognitive development. The role of PM components or sources other than traffic on cognitive development has been little explored.

**Objectives:**

We aimed to explore the role of PM sources in school air on cognitive development.

**Methods:**

A cohort of 2,618 schoolchildren (average age, 8.5 years) belonging to 39 schools in Barcelona (Spain) was followed up for a year. Children completed computerized tests assessing working memory, superior working memory, and inattentiveness during four visits. Particulate matter ≤ 2.5 μm (PM2.5) was measured during two 1-week campaigns in each school, both outdoors and in the classroom. Source apportionment resulted in nine sources: mineral, organic/textile/chalk, traffic, secondary sulfate and organics, secondary nitrate, road dust, metallurgy, sea spray, and heavy oil combustion. Differences in cognitive growth trajectories were assessed with mixed models with age-by-source interaction terms.

**Results:**

An interquartile range increase in indoor traffic-related PM2.5 was associated with reductions in cognitive growth equivalent to 22% (95% CI: 2%, 42%) of the annual change in working memory, 30% (95% CI: 6%, 54%) of the annual change in superior working memory, and 11% (95% CI: 0%, 22%) of the annual change in the inattentiveness scale. None of the other PM2.5 sources was associated with adverse effects on cognitive development.

**Conclusions:**

Traffic was the only source of fine particles associated with a reduction in cognitive development. Reducing air pollution from traffic at primary schools may result in beneficial effects on cognition.

**Citation:**

Basagaña X, Esnaola M, Rivas I, Amato F, Alvarez-Pedrerol M, Forns J, López-Vicente M, Pujol J, Nieuwenhuijsen M, Querol X, Sunyer J. 2016. Neurodevelopmental deceleration by urban fine particles from different emission sources: a longitudinal observational study. Environ Health Perspect 124:1630–1636; http://dx.doi.org/10.1289/EHP209

## Introduction

Particulate matter (PM) air pollution is known to produce adverse health effects with important consequences at the population level (Brunekreef and Holgate 2002; Lim et al. 2012; WHO 2013). Although the most well-established evidence for a deleterious role of PM concerns cardiovascular and respiratory diseases, emerging evidence suggests that PM exposure can also affect neurodevelopment and cognitive function (Block et al. 2012; WHO 2013). This is supported by animal studies showing neuroinflammation and neuropathological damage in the brain as well as alterations in learning and memory functions in response to air pollution exposure (Block et al. 2012).

Particulate matter is a complex mixture of different components originating from different sources. A better understanding of which components and sources of PM are responsible for the health effects is very important from the regulatory point of view. Using chemical speciation of PM measurements and source apportionment techniques, it is now possible to estimate the concentration attributable to different sources (e.g., traffic, biomass burning, industry, or natural sources) (Viana et al. 2008). Recent studies have examined the role of source-specific pollution on health outcomes, mostly cardiovascular and respiratory mortality or hospital admissions. Most of the evidence for harmful effects of air pollution refers to traffic-related air pollution, although the effects of other sources such coal combustion, shipping, road dust, or desert dust have also been documented (Cassee et al. 2013; Ostro et al. 2011; WHO 2013). The biological mechanisms leading to neurodevelopment effects may be different from those described for cardiovascular and respiratory effects, and the chemical composition of the particles, their size, or their surface area can play a relevant role. For example, suggested mechanisms include disruptions of the nasal and olfactory barrier and the blood–brain barrier allowing direct access of ultrafine particles to the brain. PM was seen in olfactory bulb neurons in children autopsies and PM-associated metals such as nickel (Ni) or vanadium (V) were detected in the brains of dogs (Calderón-Garcidueñas et al. 2003, 2008).

In a recent longitudinal study of schoolchildren, we reported that cognitive development over 1 year showed a slower increase among children attending schools with high traffic-related air pollution levels compared with children in less polluted schools (Sunyer et al. 2015). In that study, the air pollution markers used were nitrogen dioxide (NO_2_), elemental carbon, and ultrafine particle number. Interestingly, fine particle (PM with aerodynamic diamater ≤ 2.5 μm; PM_2.5_) mass concentrations at the studied schools were not correlated with traffic pollution, and most of the contribution to PM_2.5_ levels was due to mineral and organic sources (Amato et al. 2014). PM_2.5_ levels are the universal indicator of air quality because of their overwhelming adverse association with many health indicators (WHO 2013). Here, we aim to explore the role of all the different sources of PM_2.5_ in school air on cognitive development.

## Methods

### Design and Population

A cohort of schoolchildren in Barcelona (Spain) was followed up for a year (study period: January 2012–March 2013) as part of the BREATHE (BRain dEvelopment and Air polluTion ultrafine particles in scHool childrEn) project. Cluster sampling was performed by first selecting 40 schools and then inviting all students without special needs in grades 2 through 4 (7–10 years of age) to participate. Using a map of NO_2_ levels in the city, pairs of one high-pollution and one low-pollution school matched by socioeconomic vulnerability index (census tract–level indicator based on level of education, unemployment, and occupation) and type of school (i.e., public/private) were selected. A total of 39 schools (18 pairs and 1 trio) agreed to participate and were included in the study (Sunyer et al. 2015). Participating schools were similar to the remaining schools in Barcelona in terms of the socioeconomic vulnerability index (Sunyer et al. 2015). Families of 2,897 children (59% of those eligible) agreed to participate in the study. All parents or guardians signed the informed consent form approved by the Clinical Research Ethical Committee (No. 2010/41221/I) of the IMIM-Parc de Salut MAR, Barcelona, Spain.

### Outcomes: Cognitive Development

Cognitive development was assessed through long-term change in working memory and attention, because these functions grow steadily during pre-adolescence (Anderson 2002; Rueda et al. 2005). Children were evaluated every 3 months over four repeated visits using computerized tests. The computerized versions chosen [the *n*-back task on working memory (Anderson 2002) and the attentional network task (ANT) (Rueda et al. 2004)] were validated with brain imaging (Rueda et al. 2004; Thomason et al. 2009) and in the general population (Forns et al. 2014).

Briefly, in the *n*-back task, subjects are presented a sequence of stimuli in the screen (e.g., a number), one at a time, and they need to respond (i.e., hit a button) only when the current stimulus matches the one presented *n* steps before. In the present study, we analyzed only 2-back task as a measure of working memory and 3-back task as a measure of superior working memory, and used only the numbers stimuli, although other tests were also administered. These choices were based on good properties observed for these tests in the same cohort (e.g., clear age-dependent slope and little learning effect) (Sunyer et al. 2015). For each of these two tests, we measured detectability (d prime, *d*´), which is the normalized proportion of correctly identified targets minus the normalized proportion of false alarm hits, *d*´ = (*z* hit rate – *z* false alarm rate). A higher *d*´ indicates more accurate test performance. In ANT, subjects have to respond whether the central fish in a row is pointing to the left or right by pressing the corresponding button on the mouse. We used hit reaction time standard error (HRT-SE), a measure of response speed consistency, throughout the test (Sunyer et al. 2015). A high HRT-SE indicates highly variable reactions and is considered a measure of inattentiveness.

### Air Pollution Exposure

Air pollution measurements were taken simultaneously for each pair of schools during two 1-week periods separated by 6 months (sampling campaign 1: January–June 2012; sampling campaign 2: September 2012–February 2013). Only a pair of schools was measured each week. High-volume samplers (MCV SA, Barcelona, Spain) for particulate matter < 2.5 μm (PM_2.5_) were installed indoors in a classroom and outdoors in the playground during school hours (0900–1700 hours) from Monday through Thursday. A detailed description of the measurement campaigns and the instruments can be found elsewhere (Amato et al. 2014). Briefly, filters from samplers were divided in different pieces to determine concentrations of major and trace elements via inductively coupled plasma mass spectrometry and atomic emission spectrometry (ICP-MS and ICP-AES); concentrations of sulfate, nitrate, and chloride ions via ion chromatography (IC) and ammonium via a specific electrode; and concentrations of organic carbon (OC) and elemental carbon (EC) via a thermal-optical transmission technique (TOT).

All measurements (including indoor and outdoor measurements) were pooled to conduct the source apportionment analysis, because this was shown to provide the best results in these data (Amato et al. 2014). Source apportionment was performed using a constrained positive matrix factorization (PMF) model based on 33 chemical species. PMF is a weighted least-squares technique that allows accounting for the uncertainty associated with the analytical procedure, and was run by means of the Multilinear Engine program, which allowed the handling of *a priori* information such as the source profile of local road dust and sea spray (Amato et al. 2014). This technique returned a solution that identified nine main factors/sources responsible for the variability of PM_2.5_ mass concentrations with an *R* ^2^ of 0.95. The nine sources were identified as mineral, organic/textile/chalk, traffic (that included exhaust and non-exhaust contributions), secondary sulfate and organics, secondary nitrate, road dust (resuspended street dust), metallurgy, sea spray, and heavy oil combustion (mostly from shipping in the study area and period). The elements identifying the sources are summarized in Table 1.

**Table 1:** Main elements identifying the estimated sources.

Source	Identifying species (tracers)
Mineral	Al, Mg, Li, Fe, Ca, Ti, Rb
Traffic	EC, Cu, Sb, Sn, Fe
Organic/textile/chalk	OC, Ca, Sr
Secondary sulfate and organics	SO_4_^2–^, NH_4_^+^
Secondary nitrate	NO_3_^–^
Road dust	Ca, Fe, Cu, Sb
Metallurgy	Zn, Pb, Cd, Mn, Cu
Sea spray	Na, Cl^–^
Heavy oil combustion	V, Ni
Abbreviations: Al, aluminum; Ca, calcium; Cd, cadmium; Cl^–^, chloride ion; Cu, copper; EC, elemental carbon; Fe, iron; Li, lithium; Mg, magnesium; Mn, manganese; Na, sodium; NH_4_^+^, ammonium cation; Ni, nickel; NO_3_^–^, nitrate; OC, organic carbon; Pb, lead; Rb, rubidium; Sb, antimony; Sn, tin; SO_4_^2–^, sulfate; Sr, strontium; Ti, titanium; V, vanadium; Zn, zinc.	

Outdoor and indoor long-term total and source-specific PM levels were obtained by averaging the two 1-week measures of each school. To minimize the effect of meteorology and other seasonal effects in the results, we conducted paired statistical analyses (described below) to restrict comparisons between schools that were measured simultaneously.

### Contextual and Individual Covariates

Sociodemographic factors included questionnaire-based parents’ responses on parental education, marital status, environmental tobacco smoke at home, and a neighborhood socioeconomic status vulnerability index (Sunyer et al. 2015) calculated both at the school and home addresses. Exposure to traffic PM_2.5_ at home was estimated at the geocoded postal address using available maps based on land use regression models (Eeftens et al. 2012; Sunyer et al. 2015). Noise levels in the classroom before children arrived (as a measure of traffic-related noise) were also measured (Sunyer et al. 2015).

### Statistical Analysis

Due to the multilevel nature of the data (i.e., visits within children within schools), we used linear mixed-effects models with the four repeated cognitive parameters as outcomes and random effects for child and school. Age at each visit (centered at visit 1) was included in the model to capture the growth trajectory of the cognitive test. An interaction between age and school concentrations of individual PM sources was included to capture changes in growth trajectory associated with school air pollution exposure. The latter was the effect of interest in this study. Potential confounders were identified using directed acyclic graphs (DAG) as described elsewhere (Sunyer et al. 2015), and they included sex, maternal education (primary or less/secondary/university), residential neighborhood socioeconomic status, and air pollution exposure at home. Indicators of school pair were included in the model to restrict comparisons within pairs of schools measured during the same days, thus removing potential differences in air pollution levels between schools that were attributable to meteorology or seasonality. The model equation was the following,

*Y_psit_* = β_0p_ + β_1_(Age*_psit_* – Age*_psi1_*) + β_2_(PM_source)*_ps_* + β_3_(Age*_psit_* – Age*_psi1_*) × (PM_source)*_ps_* + **Z**η + u*_ps_* + v*_psi_* + ε*_psit_*,

where *Y_psit_* is the cognitive test result for subject *i* in school *s* (belonging to pair *p*) at visit *t*, *t* = (1,2,3,4), β_0p_ are pair-specific intercepts, **Z** is a matrix including all confounders, η is a vector of parameters associated to confounders, u*_ps_* are random effects at school level, assumed normally distributed with mean 0 and variance σ_u_^2^, v*_psi_* are random effects associated with subject *i* in school *s*, assumed normally distributed with mean 0 and variance σ_v_^2^, and ε*_psit_* are the model residuals assumed normally distributed with mean 0 and variance σ_e_^2^. Deviations from linearity were assessed with generalized additive mixed models. Analyses were repeated without the pair indicator and also with further adjustment for total PM_2.5_ levels and the interaction between age and total PM_2.5_ (Mostofsky et al. 2012). The interactions between age and maternal education (*p* > 0.15 for all outcomes) and age and socioeconomic status (*p* > 0.5 for all outcomes) were unrelated to cognitive development and were not included in the models.

Models included only PM_2.5_ concentrations from a single source at a time, and separate models were fitted for each source. Likewise, separate models were fitted for indoor and outdoor concentrations. Regression coefficients were rescaled to represent the change in the outcome associated with an interquartile range change in source-specific PM_2.5_ levels.

We also provided the results using tracers (chemical elements identifying the source) instead of sources for those PM_2.5_ sources showing significant or suggestive adverse effects on cognition. Statistical significance was set at *p* < 0.05.

## Results

Table 2 summarizes the characteristics of the selected schools with respect to the high versus low air pollution indicator used at the design stage. Schools with high air pollution showed lower area-level deprivation and less greenness, had a lower percentage of public schools and higher indoor noise levels, were closer to busy roads, and their students tended to live closer to the school. Education quality was equivalent in the two groups.

**Table 2:** Characteristics of selected schools according to the air pollution indicator used at the design stage (city map of NO_2_ levels).

Characteristic	Low air pollution	High air pollution
Number	20	19
Socioeconomic vulnerability index	0.52 ± 0.24	0.41 ± 0.16
School greenness (NDVI)	0.31 ± 0.10	0.15 ± 0.03
Public school (%)	55	42
Education quality (PISA 2012)	3.9 ± 1.3	3.9 ± 1.8
Noise level in classroom (dB)	37.2 ± 4.9	40.1 ± 5.0
Distance to busy roads (m)	369 ± 357	118 ± 178
Average distance to children home (m)	2,432 ± 2,338	1,048 ± 1,613
Abbreviations: NDVI, Normalized Difference Vegetation Index; PISA, Programme for International Student Assessment. Data are number, percent, or mean ± SD. This table is a partial reproduction of published work (Table 5 in Sunyer et al. 2015).		

The average age of participants at baseline was 8.5 years. A total of 2,618 (90.3%) children had data on the three outcomes in at least one visit. Children without data on cognitive outcomes more often attended public schools (54% vs. 33%), but there were no differences in terms of school vulnerability index (0.45 vs. 0.42). Around half of the children were girls and they attended 2nd, 3rd, and 4th grade (37%, 36%, and 27%, respectively) in the first visit. Thirty-four percent of them attended a public school, and the rest attended a private school (Table 3). More than half of the mothers (58.9%) had a university education, whereas 12.5% had at most achieved primary education. Thirty-one percent of them lived in areas of high deprivation according to the socioeconomic status vulnerability index. More details of the study population can be found elsewhere (Sunyer et al. 2015).

**Table 3:** Mean (± SD) of cognitive outcomes by characteristics of participants.

Characteristics	*n* (%)^*a*^	Working memory (WM) (2-back numbers, *d*´ × 100)		Superior WM (3-back numbers, *d*´ × 100)		Inattentiveness (HRT-SE, ms)	
		Baseline	Change	Baseline	Change	Baseline	Change
All	2,618 (100)	224 ± 126	30 ± 156	118 ± 100	20 ± 130	272 ± 90	–38 ± 89
Sex
Male	1,316 (50.3)	229 ± 129	25 ± 155	123 ± 103	16 ± 129	261 ± 89*	–35 ± 89
Female	1,302 (49.7)	220 ± 122	35 ± 157	113 ± 96	23 ± 132	284 ± 89	–41 ± 88
Type of school
Public	860 (32.8)	215 ± 129*	36 ± 149	111 ± 102*	32 ± 126*	274 ± 91	–39 ± 89
Private	1,758 (67.2)	229 ± 124	27 ± 159	121 ± 99	14 ± 132	271 ± 89	–38 ± 89
Maternal education
Primary or less	337 (12.9)	188 ± 134*	25 ± 162	82 ± 91*	25 ± 130	308 ± 88*	–40 ± 92
Secondary	743 (28.4)	213 ± 124	32 ± 160	118 ± 102	14 ± 132	274 ± 90	–31 ± 93
University	1,538 (58.7)	237 ± 122	30 ± 153	126 ± 100	21 ± 130	264 ± 88	–41 ± 85
SES vulnerability at home
Less deprived	980 (37.4)	233 ± 124*	29 ± 151	127 ± 102*	18 ± 131	265 ± 89*	–38 ± 87
Middle deprived	807 (30.8)	227 ± 125	27 ± 159	116 ± 98	27 ± 131	274 ± 92	–37 ± 91
High deprived	831 (31.7)	212 ± 128	34 ± 159	110 ± 100	15 ± 130	281 ± 88	–39 ± 89
School Pair
Low polluted	1,328	226 ± 125	36 ± 154	120 ± 100	23 ± 131	272 ± 89	–42 ± 86
High polluted	1,290	222 ± 126	24 ± 158	116 ± 100	16 ± 130	273 ± 90	–34 ± 91
Residential PM_2.5_ from traffic
1st quartile	635	224 ± 128	32 ± 157	121 ± 101	17 ± 125	275 ± 94	–38 ± 83
2nd quartile	662	229 ± 124	22 ± 159	117 ± 103	21 ± 139	272 ± 90	–38 ± 86
3rd quartile	659	224 ± 126	37 ± 150	123 ± 98	15 ± 128	271 ± 89	–41 ± 93
4th quartile	662	220 ± 125	29 ± 157	111 ± 98	25 ± 129	271 ± 86	–37 ± 92
^***a***^Number of participants with data at baseline. *p < 0.05 when testing equality between groups.							

During the 1-year follow-up, working memory increased on average by 13.0%, superior working memory by 16.5%, and inattentiveness decreased by 14% (Table 3). At baseline, lower scores were observed for girls, children attending public schools, children from mothers with low education, and children living in more deprived areas. Children from public schools showed a greater change in superior working memory over follow-up than those from private schools. Change over follow-up was not significantly associated with other characteristics in crude analyses (Table 3).

**Figure 1:**
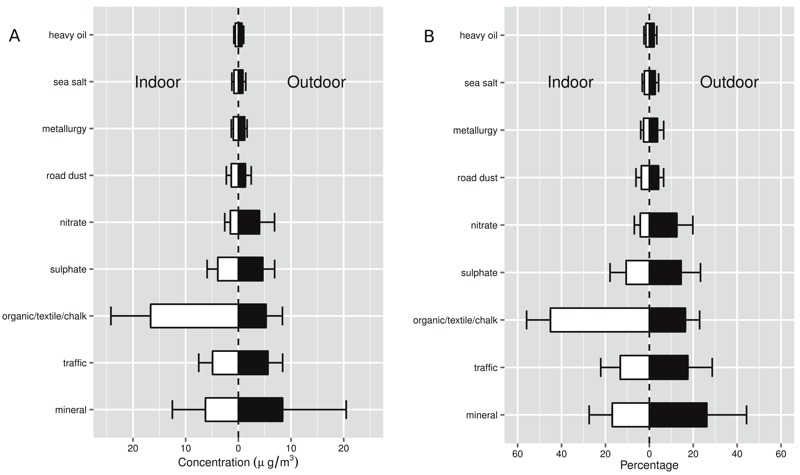
Average source concentrations (*A*) and percent of PM_2.5_ concentrations (*B*) inside (indoor) and outside (outdoor) of schools. Error bars indicate mean ± SD.

The median of school-averaged PM_2.5_ mass concentrations was 28 μg/m^3^ outdoors and 36 μg/m^3^ indoors. Mineral (27%) was the source contributing the highest concentration to outdoor PM_2.5_ levels, followed by traffic (17%), organic/textile/chalk (16%), sulfate (14%), nitrate (13%), and smaller contributions of road dust (4%), metallurgy (4%), sea salt (3%), and heavy oil combustion (2%) (Figure 1). Indoor concentrations were in general smaller and followed the same ordering than outdoor sources, except for organic/textile/chalk, with a strong indoor origin and representing the highest contribution to indoor PM_2.5_ (45%). Table 4 describes the variation of concentrations by source across schools. Mineral exhibited the largest variation, with schools below the 25th percentile having mineral concentrations that represented at most 5.5% of the total levels, whereas in schools above the 75th percentile mineral contributed > 34% of the total PM_2.5_ levels. The indoor organic/textile/chalk source also showed large variations between schools. The interquartile range for the traffic source was higher for indoor than for outdoor levels, probably reflecting the effect of class orientation on infiltration (Amato et al. 2014). More details on source apportionment results can be found elsewhere (Amato et al. 2014).

**Table 4:** Description of source contributions to PM_2.5_ in terms of mass and as a percentage of total PM_2.5_ mass.

Source	Concentration (μg/m^3^)				Percent			
	Percentile			IQR	Percentile			IQR
	25th	50th	75th		25th	50th	75th	
Outdoor PM_2.5_	22.6	28.1	35.8	13.2	—	—	—	—
Mineral	1.2	2.6	12.7	11.5	5.5	11.3	33.7	28.2
Traffic	4.1	5.2	6.8	2.7	12.9	20.5	26.1	13.3
Organic/textile/chalk	2.3	4.8	7.1	4.7	10.8	14.7	20.2	9.4
Secondary sulfate and organics	2.6	4.5	5.7	3.1	10.3	13.7	24.7	14.4
Secondary nitrate	1.9	3.2	5.1	3.2	7.1	11.3	15.6	8.5
Road dust	0.6	1.1	1.8	1.2	2.3	4.3	5.5	3.2
Metallurgy	0.9	1.2	1.5	0.6	3.1	3.9	5.4	2.3
Sea spray	0.5	0.7	1.1	0.6	1.8	2.3	3.5	1.7
Heavy oil combustion	0.5	0.6	0.8	0.4	1.6	2.2	2.8	1.1
Indoor PM_2.5_	29.2	35.6	41.5	12.3	—	—	—	—
Mineral	2.0	3.9	7.2	5.2	6.5	11.6	20.6	14.1
Traffic	3.0	4.4	6.8	3.8	9.7	12.7	20.0	10.3
Organic/textile/chalk	12.3	15.3	20.1	7.8	37.2	44.8	48.9	11.6
Secondary sulfate and organics	2.3	3.6	5.4	3.1	7.2	10.7	15.0	7.8
Secondary nitrate	0.9	1.1	1.9	1.0	2.6	3.8	5.3	2.8
Road dust	0.5	1.3	2.1	1.6	1.7	3.3	5.2	3.5
Metallurgy	0.7	0.9	1.2	0.5	2.0	2.8	3.3	1.3
Sea spray	0.6	0.7	1.0	0.5	1.7	2.0	2.7	1.1
Heavy oil combustion	0.4	0.6	0.7	0.3	1.1	1.4	2.0	0.9
IQR, interquartile range.								

Correlations between indoor and outdoor levels of the same sources were generally greater than 0.7 (see Table S1). Exceptions to this pattern were organic/textile/chalk, with indoor–outdoor correlation close to zero; road dust, with a correlation of 0.14; and to a lesser extent mineral, with a correlation of 0.64. The highest correlations between school levels of outdoor sources were in the 0.5–0.6 range, including the correlations of mineral with organic/textile/chalk, sea salt, and road dust, and the pairs metallurgy–secondary nitrate, heavy oil combustion–secondary sulfate, and road dust–organic/textile/chalk. With regard to indoor sources, the pairs of sources mentioned above for outdoor levels showed similarly high correlations, whereas secondary nitrate also showed high correlations with secondary sulfate and heavy oil combustion, and traffic showed a negative correlation of –0.52 with road dust (see Table S2).

**Figure 2:**
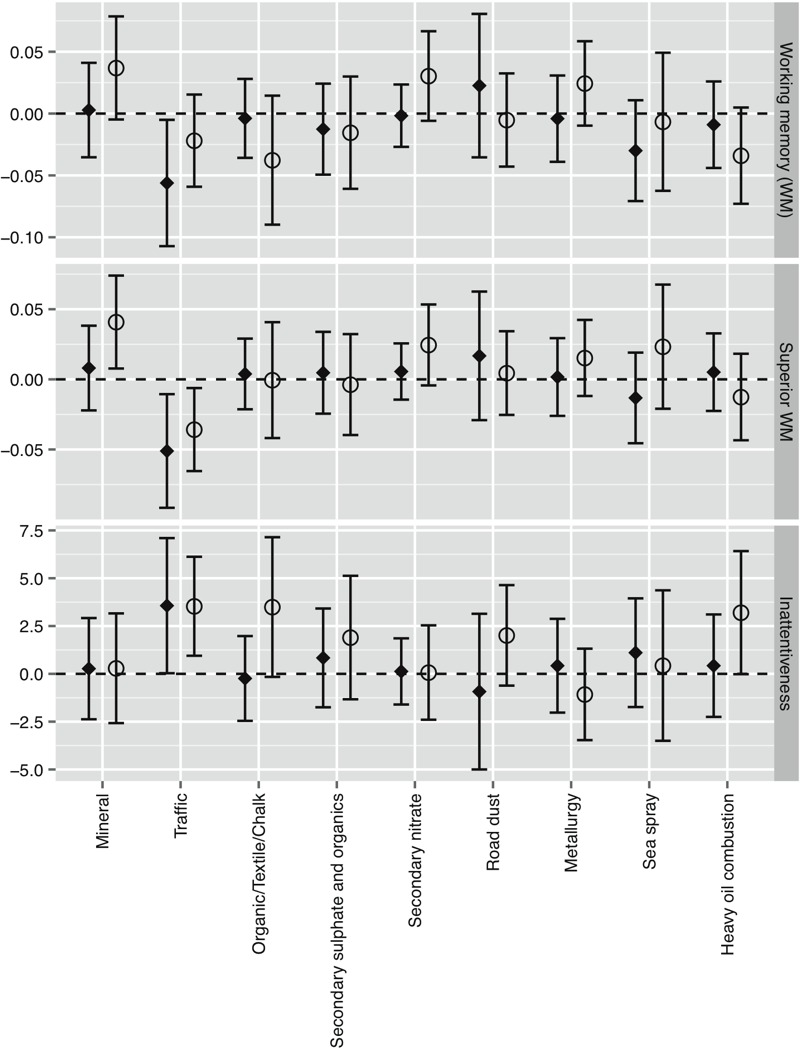
Change (95% CI) in cognitive growth per interquartile range increase in school source-specific PM_2.5_ mass concentrations. Models were adjusted for age, sex, maternal education, residential neighborhood socioeconomic status, residential PM_2.5_ levels from traffic and school pair; school and subject included as nested random effects. Working memory measured with 2-back Numbers, *d*´ × 100. Superior working memory measured with 3-back numbers, *d*´ × 100. Inattentiveness measures with HRT-SE, ms. Black diamonds (♦): indoor concentrations; open circles (o): outdoor concentrations.

Figure 2 (see also Table S3) displays the change in cognitive outcomes over the follow-up period for an interquartile range increase in source-specific PM_2.5_ concentrations. Results in unadjusted analyses were fairly similar to adjusted ones (see Table S3). Changes from the first to the third quartile in the indoor traffic source were associated with a significant reduction in working memory of –5.6 [95% confidence interval (CI): –10.7, –0.5], equivalent to 22% of the annual change experienced by the participants (see Table S3); a reduction of superior working memory of –5.1 (95% CI: –9.2, –1.1), equivalent to 30% of the annual change; and an increase of 3.6 (95% CI: 0.0, 7.1) in inattentiveness scale, equivalent to 11% of the annual change. Associations were smaller for outdoor concentrations of traffic PM_2.5_, although results were still significant for superior working memory and inattentiveness. No significant associations were found for PM_2.5_ mass concentrations from other sources, except for a positive association for outdoor concentrations of PM_2.5_ from mineral origin and superior working memory. Outdoor levels of PM_2.5_ from heavy oil combustion showed deleterious effects on inattentiveness and working memory (*p* = 0.05 and 0.09, respectively). No important deviations from linearity were detected (data not shown). When analyses were repeated excluding the school pair indicator or adjusting by environmental tobacco smoke exposure at home or traffic-related noise at school results were almost the same (data not shown). Further adjustment for total PM_2.5_ levels produced only minimal changes in the results (see Table S4).

Figure S1 provides the results when using the tracers for traffic, organic/textile/chalk, secondary nitrate, and heavy oil combustion. EC was significantly associated with all outcomes, while copper (Cu) and antimony (Sb) showed significant associations only for inattentiveness. No significant associations were found for tin (Sn), iron (Fe), nitrate (NO_3_^–^), V, Ni, OC, calcium (Ca) or strontium (Sr).

## Discussion

In a longitudinal study assessing cognitive development of schoolchildren during 1 year, we found that children attending schools with high levels of traffic-related PM_2.5_ showed a slower cognitive development. None of the other PM_2.5_ sources (mineral, organic/textile/chalk, sulfate, nitrate, road dust, metallurgy, and sea spray) showed a deleterious association with cognitive development, although associations for heavy oil combustion were also suggested. Associations with traffic pollution were stronger when considering indoor levels and these associations were detected for working memory, superior working memory, and inattentiveness. These results suggest that fine particles from traffic may produce neurotoxic effects, and that exposure to such particles at primary schools can result in a deceleration of cognitive development.

Our previous study was the first to relate primary school levels of air pollution to cognitive development (growth) in a longitudinal setting (Sunyer et al. 2015). A few other studies have related air pollution exposure at schools to neurobehavioral function at a single point in time (van Kempen et al. 2012; Wang et al. 2009), although others found no association (Clark et al. 2012). Other cross-sectional studies related personal or residential air pollution exposure with cognitive outcomes, and most of them reported positive associations (Chiu et al. 2013; Franco Suglia et al. 2008; Grahame et al. 2014; Guxens et al. 2014; Guxens and Sunyer 2012; Perera et al. 2012). Our study is the first to perform source apportionment of PM and examine the relationship of each individual source with cognitive development in children. In our study, PM from traffic was the only source associated with a slower cognitive development, which agrees with our previously published result on the effects of EC in this same cohort (Sunyer et al. 2015). Although the traffic source includes also non-exhaust particles, the correlation of the source with EC was 0.89. Most of the previous studies used markers of traffic air pollution such as EC or black carbon (BC), NO_2_, PM_2.5_ absorbance, or polycyclic aromatic hydrocarbons (PAHs) (Chiu et al. 2013; Franco Suglia et al. 2008; Grahame et al. 2014; Guxens et al. 2014; Guxens and Sunyer 2012; Perera et al. 2012).

The role of PM sources other than traffic on cognitive development has been little explored, although some studies exist on industrial pollution. An ecological study in Michigan (USA) found an increased percentage of school failure in schools with higher levels of industrial pollution (Mohai et al. 2011). Other studies in children have found that manganese (Mn) concentrations from mining and industry were associated with impaired verbal intellectual function and motor skills (Lucchini et al. 2012). In our study area, air pollution from industry sources was low, which may be the reason why we did not find associations with this source. Besides, industry emissions depend strongly on industry type, so associations are expected to vary by study setting. Our results in relation to heavy oil combustion were inconclusive. We found an association of outdoor levels of heavy oil combustion with two of the outcomes, but chance could not be excluded due to the *p*-values at the limit of significance and the lack of association with indoor levels or with Ni and V, the main elements defining this source. Unexpectedly, we found that exposure to mineral particles was beneficial for superior working memory. This finding was not observed for indoor levels and we do not know of other studies that investigated the link between mineral particles and cognitive development. This result could be a chance finding. Schools with higher mineral concentrations had sandy playgrounds, so an alternative explanation is that they also have more greenness, which can have beneficial effects on cognitive development (Dadvand et al. 2015). However, further adjustment for greenness did not change the mineral results (data not shown).

When examining the effects of chemical elements (tracers of sources) on cognitive development, the most consistent results were found for EC. Cu and Sb were significantly associated only with inattentiveness. There is still debate on which specific components linked to traffic produce health effects, but there seems to be some consensus in that the health effects are not produced by EC alone, but by other co-emissions such as semi-volatile organic compounds (SVOCs) and PAHs that are adsorbed onto the EC core (Grahame et al. 2014). In our study, we also found some suggestions for deleterious effects of particles from heavy oil combustion, which goes along with the hypothesis of particles from combustion being harmful for the brain. Thus, our findings of slower cognitive development associated with exposure to EC may have implications beyond the effects of traffic emissions. For example, biomass burning can also be an important source of EC/BC and PAHs (Grahame et al. 2014), and the high concentrations of indoor pollution from biomass burning in developing countries could have important effects on the cognitive development of exposed children. In our study area, PM mass concentrations from biomass burning were negligible and this question could not be investigated (Reche et al. 2012).

Toxicological studies support the neurotoxic effects of motor exhaust particles (Grahame et al. 2014). The main biological mechanisms involve proinflammatory and inflammatory effects in the brain following brain deposition of particles or as a result of systemic inflammation produced by deposition of particles in the respiratory tract and alteration of blood–brain barrier function (Block et al. 2012; Calderón-Garcidueñas et al. 2008; Grahame et al. 2014). The brain may be especially vulnerable to oxidative stress, and diesel particles (highly enriched in EC) have been shown to activate microglia, which can produce neurotoxicity via oxidative stress (Block et al. 2012; Grahame et al. 2014). Fine particles from other vehicle sources such as brakes could also contribute to the effects beyond motor exhaust, given the association observed for elements generated by brakes abrasion with inattentiveness in our study and their established potential neurotoxicity (Bandmann et al. 2015). Ni and V, which could also lead to oxidative stress, were not associated with cognition in our study.

Our study had several strengths, including its longitudinal design with repeated outcome measurements and the direct measurements of air pollution both indoors and outdoors at schools. The study also had some limitations, such as a relatively small number of schools and the possibility of residual confounding by socioeconomic characteristics. The latter was extensively explored in our previous study, and all analyses suggested the observed effects were not attributable to residual confounding (Sunyer et al. 2015). In our analyses, schools were matched by socioeconomic characteristics and type of school, thus reducing potential differences, and although children from more educated families attended schools with lower air pollution levels, differences were small. Because of the observational nature of the study, it cannot be ruled out that children attending schools with high levels of pollution shared other unmeasured characteristics (e.g., not captured socioeconomic dimensions, different level of social interaction) that affected their cognitive development. Differences in cognition were already present at the beginning of the study. This would still be consistent with our hypotheses, because the cognitive functions studied were already developing in the previous years and children were already exposed to school air pollution. Importantly, we observed that these differences widened during the study period, but we could not ignore that children of more-polluted schools were already in a slower cognitive trajectory because of early-life exposures or socioeconomic factors.

Air pollution levels were based on direct measurements at schools on two different seasons. Although this may represent an improvement over previous papers, which used models to estimate air pollution concentrations at schools, our estimations were still imperfect estimates of annual concentrations. This measurement error is unlikely to be related to school characteristics, in which case it would bias the results toward the null. Another limitation was that, in order to increase the statistical power of source apportionment, this was applied to the joint set of all indoor and outdoor measurements, which may generate some artifacts in source identification. Our data could also be affected by other issues in source estimation, such as imperfect separation of road dust and the mineral sources. Uncertainty in source estimation, if properly accounted for, would widen our confidence intervals (Kioumourtzoglou et al. 2014). We did not have data on other pollutants such as gases or volatile compounds that may be related to cognitive effects, but these are expected to be correlated with the estimated sources. Further studies in different settings are needed to assess the generalizability of these results. Finally, it is worth mentioning that we examined working memory and inattentiveness, but not other domains of cognition such as visuospatial ability or language.

## Conclusions

This study aimed to investigate whether levels of PM at schools were associated with cognitive development separately for each PM source. We found that levels of PM from traffic were associated with important reductions in cognitive growth over a 1-year period in primary school children. Future studies should examine whether the effects observed at primary school age are long-lasting and have consequences over the life course.

## Supplemental Material

(237 KB) PDF
