# Supplementary material for: Neurodevelopmental Deceleration by Urban Fine Particles from Different Emission Sources: A Longitudinal Observational Study
Source: Environ Health Perspect. 2016 Apr 29;124(10):1630–6. doi: 10.1289/EHP209 (PMC5047777; doi:10.1289/EHP209)
Supplement: (237 KB) PDF [file EHP209.s001.acco.pdf]

Environ Health Perspect DOI: 10.1289/EHP209
Advance Publication: Not Copyedited

Note to readers with disabilities: *EHP* strives to ensure that all journal content is accessible to all readers. However, some figures and Supplemental Material published in *EHP* articles may not conform to 508 standards due to the complexity of the information being presented. If you need assistance accessing journal content, please contact ehp508@niehs.nih.gov. Our staff will work with you to assess and meet your accessibility needs within 3 working days.

Supplemental Material

Neurodevelopmental Deceleration by Urban Fine Particles from Different Emission Sources: A Longitudinal Observational Study

Xavier Basagaña, Mikel Esnaola, Ioar Rivas, Fulvio Amato, Mar Alvarez-Pedrerol, Joan Forns, Mònica López-Vicente, Jesús Pujol, Mark Nieuwenhuijsen, Xavier Querol, and Jordi Sunyer

Table of Contents

Table S1. Correlation between outdoor source-specific mass concentrations. The main diagonal (in bold font) includes the indoor-outdoor correlation for a fixed source.

Table S2. Correlation between indoor source-specific mass concentrations. The main diagonal (in bold font) includes the indoor-outdoor correlation for a fixed source.

Table S3. Change (95% confidence interval) in cognitive growth per interquartile range increase in school source-specific PM2.5 mass concentrations, with and without adjustment.

Table S4. Change (95% confidence interval) in cognitive growth per interquartile range increase in school source-specific PM2.5 mass concentrations, without adjustment for total $PM_{2.5}$ levels (original model) and with adjustment for total $PM_{2.5}$ levels

Figure S1. Change (95% confidence interval) in cognitive growth per interquartile range increase in concentrations of elements defining the several sources (N= 2618). Models were adjusted for age, sex, maternal education, residential neighbourhood socio-economic status, residential $PM_{2.5}$ levels from traffic and school pair; school and subject included as nested random effects. Working memory measured with 2-back Numbers, d'*100. Superior working memory measured with 3-back Numbers, d'*100. Inattentiveness measures with HRT-SE, ms. Black diamonds (♦): indoor concentrations; Empty circles (o): outdoor concentrations

Table S1. Correlation between outdoor source-specific mass concentrations. The main diagonal (in bold font) includes the indoor-outdoor correlation for a fixed source.

Sources	$PM_{2.5}$	Mineral	Traffic	Organic/ Textile/ Chalk	Sulphate	Nitrate	Road dust	Metallurgy	Sea salt	Heavy oil combustion
$PM_{2.5}$	**0.06**									
Mineral	0.85	**0.64**								
Traffic	0.07	-0.23	**0.73**							
Organic/Textile/Chalk	0.76	0.58	-0.02	**0.03**						
Sulphate	0.04	-0.39	0.29	-0.43	**0.91**					
Nitrate	0.52	0.15	0.26	0	0.31	**0.71**				
Road dust	0.64	0.57	-0.04	0.61	-0.2	0.14	**0.14**			
Metallurgy	0.16	-0.13	0.23	-0.16	0.3	0.56	-0.41	**0.81**		
Sea salt	0.51	0.56	-0.29	0.43	-0.09	0.09	0.13	-0.14	**0.69**	
Heavy Oil combustion	0.25	0.01	0.04	-0.03	0.55	0.22	-0.25	0.26	-0.11	**0.76**

Table S2. Correlation between indoor source-specific mass concentrations. The main diagonal (in bold font) includes the indoor-outdoor correlation for a fixed source.

Sources	$PM_{2.5}$	Mineral	Traffic	Organic/ Textile/ Chalk	Sulphate	Nitrate	Road dust	Metallurgy	Sea salt	Heavy oil combustion
$PM_{2.5}$	**0.06**									
Mineral	0.82	**0.64**								
Traffic	-0.28	-0.41	**0.73**							
Organic/Textile/Chalk	0.91	0.65	-0.39	**0.03**						
Sulphate	-0.29	-0.25	0.12	-0.10	**0.91**					
Nitrate	0.07	-0.19	0.22	0.28	0.54	**0.71**				
Road dust	0.52	0.48	-0.52	0.54	-0.49	-0.38	**0.14**			
Metallurgy	-0.14	-0.23	0.31	0.06	0.48	0.54	-0.08	**0.81**		
Sea salt	0.53	0.57	-0.28	0.48	-0.42	-0.08	0.15	-0.2	**0.69**	
Heavy Oil combustion	0.06	-0.07	0.12	0.25	0.64	0.58	0.17	0.43	0.18	**0.76**

Table S3. Change (95% confidence interval) in cognitive growth per interquartile range increase in school source-specific PM2.5 mass concentrations, with and without adjustment.

	Working memory (WM)[a]		Superior WM[b]		Inattentiveness[c]	
	Unadjusted	Adjusted[d]	Unadjusted	Adjusted[d]	Unadjusted	Adjusted[d]
Overall change	26 (21, 32)	26 (20, 32)	17 (13, 22)	17 (12, 22)	-33 (-36, -30)	-33 (-36, -30)
Indoor						
Mineral	-0.6 (-4.3, 3.1)	0.3 (-3.5, 4.1)	0.1 (-2.8, 3.0)	0.8 (-2.2, 3.8)	0.7 (-1.9, 3.3)	0.3 (-2.4, 2.9)
Traffic	-5.2 (-10.1, -0.2)*	-5.6 (-10.7, -0.5)*	-4.0 (-7.9, 0.0)*	-5.1 (-9.2, -1.1)*	3.2 (-0.3, 6.6)	3.6 (0.0, 7.1)
Organic/Textile/Chalk	-0.4 (-3.5, 2.7)	-0.4 (-3.6, 2.8)	0.6 (-1.9, 3.1)	0.4 (-2.1, 2.9)	-0.4 (-2.6, 1.8)	-0.2 (-2.5, 2.0)
Secondary sulphate and organics	-2.6 (-6.1, 0.9)	-1.3 (-4.9, 2.4)	-0.8 (-3.6, 1.9)	0.5 (-2.4, 3.4)	1.6 (-0.9, 4.1)	0.8 (-1.7, 3.4)
Secondary nitrate	-0.8 (-3.3, 1.6)	-0.2 (-2.7, 2.4)	0.2 (-1.8, 2.1)	0.6 (-1.4, 2.6)	0.4 (-1.3, 2.2)	0.1 (-1.6, 1.9)
Road dust	2.7 (-3.0, 8.5)	2.3 (-3.5, 8.1)	1.2 (-3.3, 5.8)	1.7 (-2.9, 6.3)	-0.7 (-4.8, 3.3)	-0.9 (-5.0, 3.1)
Metallurgy	-1.5 (-4.9, 1.9)	-0.4 (-3.9, 3.1)	0.0 (-2.6, 2.7)	0.2 (-2.6, 2.9)	0.9 (-1.5, 3.3)	0.4 (-2.0, 2.9)
Sea spray	-2.6 (-6.6, 1.3)	-3.0 (-7.1, 1.1)	-1.1 (-4.2, 2.0)	-1.3 (-4.6, 1.9)	0.7 (-2.1, 3.5)	1.1 (-1.7, 3.9)
Heavy oil combustion	-2.9 (-6.3, 0.4)	-0.9 (-4.4, 2.6)	-0.6 (-3.2, 2.1)	0.5 (-2.3, 3.3)	1.2 (-1.4, 3.8)	0.4 (-2.3, 3.1)
Outdoor						
Mineral	5.2 (1.2, 9.3)*	3.7 (-0.5, 7.9)	4.7 (1.4, 7.9)*	4.1 (0.8, 7.4)*	-0.8 (-3.7, 2.0)	0.3 (-2.6, 3.2)
Traffic	-1.8 (-5.3, 1.8)	-2.2 (-5.9, 1.5)	-2.4 (-5.2, 0.4)	-3.6 (-6.5, -0.6)*	3.4 (0.9, 5.9)*	3.5 (0.9, 6.1)*
Organic/Textile/Chalk	-2.4 (-7.6, 2.7)	-3.8 (-9.0, 1.4)	0.8 (-3.2, 4.9)	-0.1 (-4.2, 4.1)	2.7 (-0.9, 6.4)	3.5 (-0.2, 7.1)
Secondary sulphate and organics	-3.1 (-7.5, 1.2)	-1.5 (-6.1, 3.0)	-1.9 (-5.4, 1.5)	-0.4 (-4.0, 3.2)	3.3 (0.1, 6.4)*	1.9 (-1.3, 5.1)
Secondary nitrate	1.4 (-2.2, 4.9)	3.0 (-0.6, 6.7)	1.6 (-1.2, 4.4)	2.5 (-0.4, 5.3)	0.6 (-1.9, 3.0)	0.1 (-2.4, 2.5)
Road dust	0.5 (-3.1, 4.2)	-0.5 (-4.3, 3.2)	1.1 (-1.8, 4.0)	0.4 (-2.5, 3.4)	0.9 (-1.7, 3.5)	2.0 (-0.6, 4.6)
Metallurgy	1.6 (-1.7, 5.0)	2.4 (-1.0, 5.9)	1.8 (-0.8, 4.4)	1.5 (-1.2, 4.2)	-0.9 (-3.3, 1.4)	-1.1 (-3.5, 1.3)
Sea spray	0.1 (-5.3, 5.6)	-0.7 (-6.2, 4.9)	1.8 (-2.6, 6.1)	2.3 (-2.1, 6.8)	-0.1 (-4.0, 3.8)	0.4 (-3.5, 4.4)
Heavy oil combustion	-3.2 (-7.0, 0.7)	-3.4 (-7.3, 0.5)	-1.2 (-4.2, 1.9)	-1.3 (-4.3, 1.8)	3.4 (0.3, 6.6)*	3.2 (0.0, 6.4)

[a] 2-back Numbers, d'*100
[b] 3-back Numbers, d'*100
[c] HRT-SE, ms
[d] Results as presented in Figure 2.
* p-value < 0.05 when testing that the effect of the source is equal to zero

Table S4. Change (95% confidence interval) in cognitive growth per interquartile range increase in school source-specific PM2.5 mass concentrations, without adjustment for total $PM_{2.5}$ levels (original model) and with adjustment for total $PM_{2.5}$ levels

	Working memory (WM)[a]		Superior WM[b]		Inattentiveness[c]	
	Original[d]	With PM_{25}[e]	Original[d]	With PM_{25}[e]	Original[d]	With PM_{25}[e]
Overall change	26 (20, 32)	-	17 (12, 22)	-	-33 (-36, -30)	-
Indoor						
Mineral	0.3 (-3.5, 4.1)	0.1 (-3.7, 4.0)	0.8 (-2.2, 3.8)	0.9 (-2.2, 3.9)	0.3 (-2.4, 2.9)	0.5 (-2.2, 3.2)
Traffic	-5.6 (-10.7, -0.5)*	-5.7 (-11.1, -0.4)*	-5.1 (-9.2, -1.1)*	-5.7 (-9.9, -1.4)*	3.6 (0.0, 7.1)	3.3 (-0.4, 7.0)
Organic/Textile/Chalk	-0.4 (-3.6, 2.8)	-0.4 (-3.6, 2.8)	0.4 (-2.1, 2.9)	0.4 (-2.1, 2.9)	-0.2 (-2.5, 2.0)	-0.2 (-2.4, 2.0)
Secondary sulphate and organics	-1.3 (-4.9, 2.4)	-1.2 (-4.9, 2.5)	0.5 (-2.4, 3.4)	0.5 (-2.5, 3.4)	0.8 (-1.7, 3.4)	0.7 (-2, 3.3)
Secondary nitrate	-0.2 (-2.7, 2.4)	-0.1 (-2.7, 2.4)	0.6 (-1.4, 2.6)	0.6 (-1.5, 2.6)	0.1 (-1.6, 1.9)	0.0 (-1.7, 1.8)
Road dust	2.3 (-3.5, 8.1)	2.2 (-3.6, 8.0)	1.7 (-2.9, 6.3)	1.7 (-2.9, 6.3)	-0.9 (-5.0, 3.1)	-0.8 (-4.8, 3.3)
Metallurgy	-0.4 (-3.9, 3.1)	-0.4 (-3.9, 3.1)	0.2 (-2.6, 2.9)	0.2 (-2.6, 2.9)	0.4 (-2.0, 2.9)	0.4 (-2.1, 2.9)
Sea spray	-3.0 (-7.1, 1.1)	-3.3 (-7.5, 0.8)	-1.3 (-4.6, 1.9)	-1.3 (-4.6, 2.0)	1.1 (-1.7, 3.9)	1.5 (-1.4, 4.4)
Heavy oil combustion	-0.9 (-4.4, 2.6)	-0.9 (-4.4, 2.6)	0.5 (-2.3, 3.3)	0.5 (-2.3, 3.3)	0.4 (-2.3, 3.1)	0.3 (-2.4, 3.0)
Outdoor						
Mineral	3.7 (-0.5, 7.9)	3.6 (-0.5, 7.8)	4.1 (0.8, 7.4)*	4.1 (0.8, 7.4)*	0.3 (-2.6, 3.2)	0.4 (-2.5, 3.3)
Traffic	-2.2 (-5.9, 1.5)	-2.1 (-5.9, 1.7)	-3.6 (-6.5, -0.6)*	-3.8 (-6.8, -0.8)*	3.5 (0.9, 6.1)*	3.4 (0.7, 6.1)*
Organic/Textile/Chalk	-3.8 (-9.0, 1.4)	-3.7 (-8.9, 1.5)	-0.1 (-4.2, 4.1)	-0.1 (-4.2, 4.1)	3.5 (-0.2, 7.1)	3.5 (-0.2, 7.1)
Secondary sulphate and organics	-1.5 (-6.1, 3.0)	-1.5 (-6.0, 3.1)	-0.4 (-4.0, 3.2)	-0.4 (-4.0, 3.2)	1.9 (-1.3, 5.1)	1.7 (-1.5, 5.0)
Secondary nitrate	3.0 (-0.6, 6.7)	3.1 (-0.5, 6.7)	2.5 (-0.4, 5.3)	2.5 (-0.4, 5.3)	0.1 (-2.4, 2.5)	0.0 (-2.5, 2.5)
Road dust	-0.5 (-4.3, 3.2)	-0.5 (-4.2, 3.3)	0.4 (-2.5, 3.4)	0.4 (-2.6, 3.4)	2.0 (-0.6, 4.6)	1.9 (-0.7, 4.6)
Metallurgy	2.4 (-1.0, 5.9)	2.4 (-1.0, 5.8)	1.5 (-1.2, 4.2)	1.5 (-1.2, 4.2)	-1.1 (-3.5, 1.3)	-1.1 (-3.4, 1.3)
Sea spray	-0.7 (-6.2, 4.9)	-0.9 (-6.6, 4.8)	2.3 (-2.1, 6.8)	2.4 (-2.0, 6.9)	0.4 (-3.5, 4.4)	0.8 (-3.2, 4.8)
Heavy oil combustion	-3.4 (-7.3, 0.5)	-3.4 (-7.3, 0.5)	-1.3 (-4.3, 1.8)	-1.3 (-4.3, 1.8)	3.2 (0.0, 6.4)	3.1 (-0.2, 6.3)

[a] 2-back Numbers, d'*100

[b] 3-back Numbers, d'*100

[c] HRT-SE, ms

[d] Results as presented in Figure 2

[e] Results of a model that further includes the terms $PM_{2.5}$ and age*$PM_{2.5}$

* p-value < 0.05 when testing that the effect of the source is equal to zero

Figure Legend

Figure S1. Change (95% confidence interval) in cognitive growth per interquartile range increase in concentrations of elements defining the several sources (N= 2618). Models were adjusted for age, sex, maternal education, residential neighbourhood socio-economic status, residential $PM_{2.5}$ levels from traffic and school pair; school and subject included as nested random effects. Working memory measured with 2-back Numbers, d'*100. Superior working memory measured with 3-back Numbers, d'*100. Inattentiveness measures with HRT-SE, ms. Black diamonds (♦): indoor concentrations; Empty circles (o): outdoor concentrations.

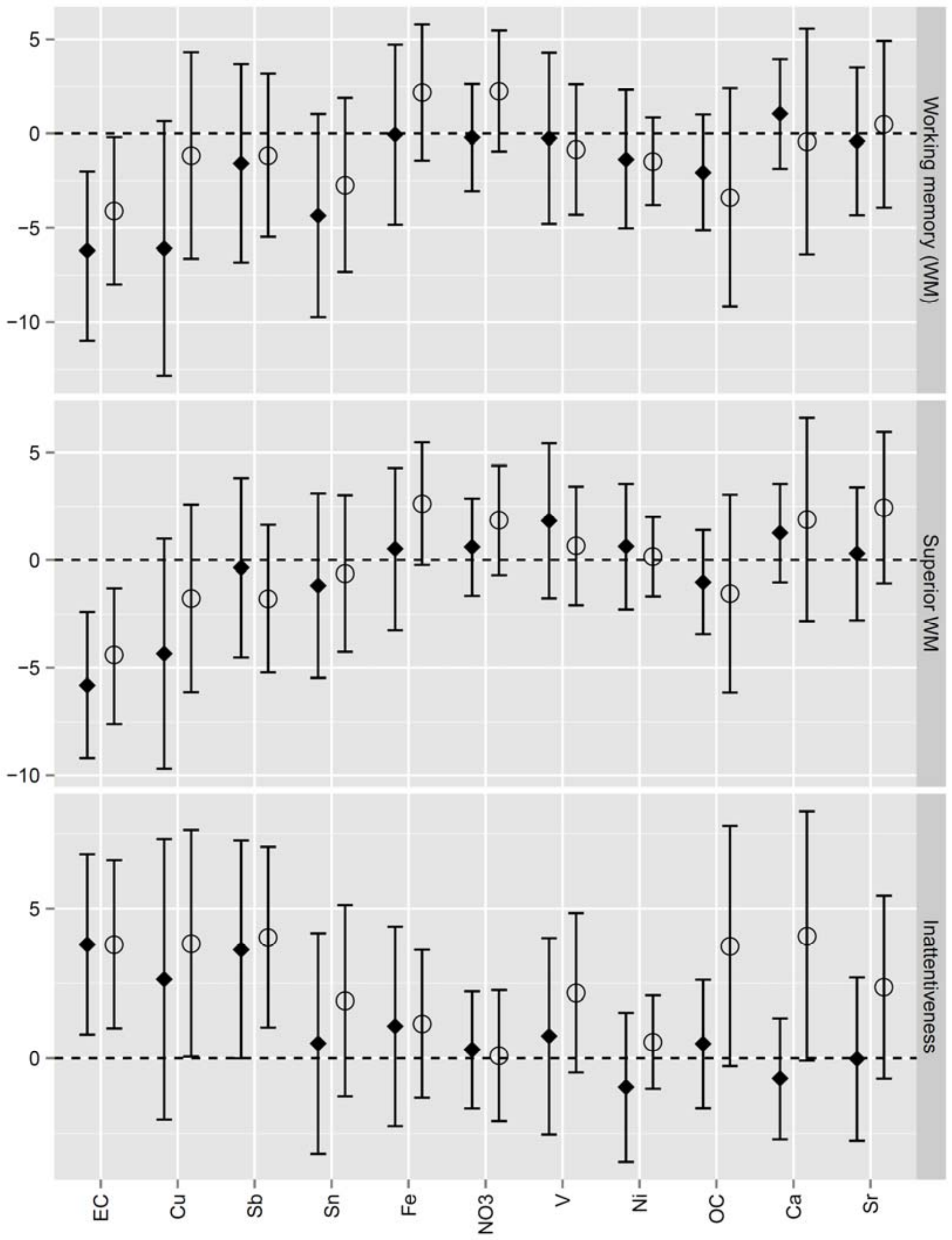

Working memory (WM)
Superior WM
Inattentiveness
5
0
-5
-10
EC
Cu
Sb
Sn
Fe
NO3
V
Ni
OC
Ca
Sr
